# Observations on Detonation Growth of Lead Azide at Microscale

**DOI:** 10.3390/mi13030451

**Published:** 2022-03-16

**Authors:** Yunfei Mu, Wei Zhang, Ruiqi Shen, Yinghua Ye

**Affiliations:** 1 School of Chemistry and Chemical Engineering, Nanjing University of Science and Technology, Nanjing 210094, China; muyunfei@njust.edu.cn (Y.M.); yyinghua@njust.edu.cn (Y.Y.); 2 Micro-Nano Energetic Devices Key Laboratory of MIIT, Nanjing 210094, China; 3 Institute of Space Propulsion, Nanjing University of Science and Technology, Nanjing 210094, China

**Keywords:** lead azide, detonation growth, photon Doppler velocimetry, polyvinylidene fluoride gauges

## Abstract

Lead azide (LA) is a commonly used primary explosive, the detonation growth of which is difficult to study because it is so sensitive and usually has a small charge size in applications. We used photon Doppler velocimetry (PDV) and calibrated polyvinylidene fluoride (PVDF) gauges to reveal the detonation growth in LA, which was pressed in the confinements with controlled heights. The particle-velocity profiles, output pressure, unsteady detonation velocity, reaction time, and reaction-zone width were obtained and analyzed. Three phases of detonation propagation of LA microcharges are discussed. The volume reactions occur at the beginning of detonation in LA microcharges without forming complete shock profiles. Then the shock front is fast with a slow chemistry reaction zone, which is compressed continuously between the height of 0.8 mm and 2.5 mm. Finally, the steady detonation is built at a height of 2.5 mm. The stable detonation velocity and *CJ* pressure are 4726 ± 8 m/s and 17.12 ± 0.22 GPa. Additionally, the stable reaction zone time and width are 44 ± 7 ns and 148 ± 11 μm. The detailed detonation process has not previously been quantified in such a small geometry.

## 1. Introduction

The detonation growth of explosives has always been the focus and hotspot of detonation and shockwave physics [1,2], which possesses great value for understanding the detonation-reaction mechanism and building reaction models for computer simulations [3]. Although extensive research has been carried out on detonations with large size, few studies which adequately cover the phenomenon at microscale exist, resulting in a lack of novel insights into the miniaturization of pyrotechnics [4,5]. To observe those microdetonations nowadays, various tabletop explosion experiments have been designed with ultrafast diagnostics or advanced techniques [6,7,8]. 

For primary explosives, such as lead azide (LA), the run distance to detonation and critical diameter are much smaller than in high explosives, showing the potential for microelectro-mechanical system (MEMS) and shock-initiation trains [5,9,10]. So far, however, there has been little discussion about the detonation growth mechanism of LA in microgeometry. Much of the research up until now has been descriptive in nature; for example, little data has been published on the deflagration-to-detonation transition (DDT) process of LA. Those data are fundamental to understanding the mechanism of microdetonation or applications in future pyrotechnics. In this paper, we attempted to reveal the nature of how detonation propagates, the detailed parameters involved, such as unsteady detonation velocity, reaction time, and reaction-zone width, and their dependence on charge height, with particle-velocity profiles measured by PDV and output pressure measured by calibrated PVDF gauges. 

## 2. Materials and Methods

### 2.1. Electric Probe Test

Detonation velocity is one of the key parameters in theorical calculations [11]. Several techniques have been developed for detonation-velocity measurement in the laboratory, such as the high-speed photograph [10], electric probe, optical fiber [11], etc. Most of these approaches were adapted to detonations of regular size and may not be effective in detonations of small charges. However, in this paper, the electric probes were still chosen to measure the steady detonation velocity of LA microcharges for their simplicity and efficiency in detecting electromagnetic signals of detonation wave fronts [12]. 

**Figure 1:**
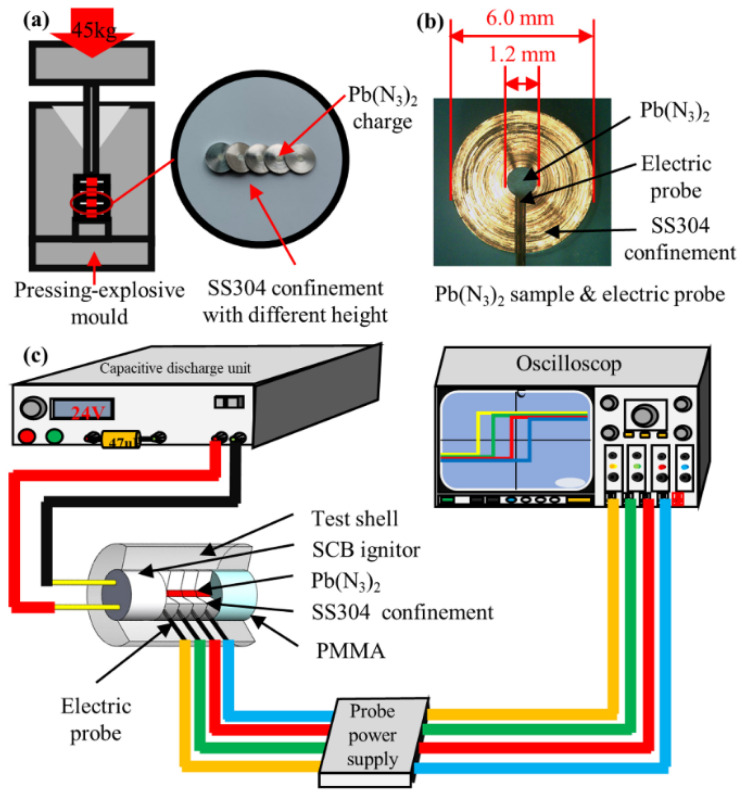
(**a**) Pressing-explosive mold and SS304 confinements. (**b**) Micrograph of LA charge, confinement and electric probe. (**c**) Schematic of experiments using electric probes to measure the detonation velocity of LA microcharges.

The LA charges, installation of active electric probes and velocity experimental apparatus are shown in Figure 1. In this paper, we used pure LA powder with a particle size of 5~15 μm, which was synthesized and refined in our laboratory. The pressed LA micropellets, with a density of 3.571 g/cm^3^ and a diameter of 1.2 mm, were placed in 304 stainless steel (SS304) confinements with a diameter of 6.0 mm and heights of 0.3, 0.5, 1.0, and 2.0 mm. The LA samples were ignited by a semiconductor bridge (SCB) igniter with a capacitive discharge unit. Four sets of electric probes with a diameter of 0.1 mm were installed in one shot. The average velocity between two sets of probes was calculated according to Equations (1) and (2). The average detonation velocity distribution can be obtained by combing different heights of confinements or placing electric probes in various positions. This is expressed as

(1) $$\bar{V}=\frac{H}{\Delta t}$$

(2) $$\Delta t=t_{1}-t_{2}$$

where $\bar{V}$ represents the average detonation velocity between two sets of electric probes, H represents the distance between two probes, Δt represents the time interval between probes, and $t_{1}$, $t_{2}$ represent the time for detonation reaching the probes.

In probe experiments, the maximum height of LA microcharges was 8.0 mm, and the probes were mounted at the heights of 0, 0.5, 1.0, 2.0, 4.0, 6.0 and 8.0 mm. Data were obtained in all cases with a minimum of three shots.

### 2.2. PVDF Gauges Test

The detonation pressure of condensed explosives has always been a fundamental parameter in detonation theory [13]. Due to the high pressure, high temperature, and destruction of detonation products, the direct measurement of detonation pressure is difficult to perform. At present, several measurements can be applied to detonation or shock-pressure tests at regular size [13,14]. Experimental determination of detonation pressure with microgeometries is, however, not widespread; most determinations are calculated based on data at the macroscale rather than measured. In this research, we depict a simple way to measure the output pressure of LA microcharges with PVDF gauges, which have lots of advantages such as a high piezoelectric coefficient, wide frequency response, low cost for mass production, no need for an extra power supply, etc. [15,16,17]. To increase the reliability of data, before use, the dynamic piezoelectric coefficient of PVDF gauges is calibrated by split Hopkinson pressure bar (SHPB) experiments, which are considered an ideal approach to determine the dynamic behavior of materials [15,18,19].

**Figure 2:**
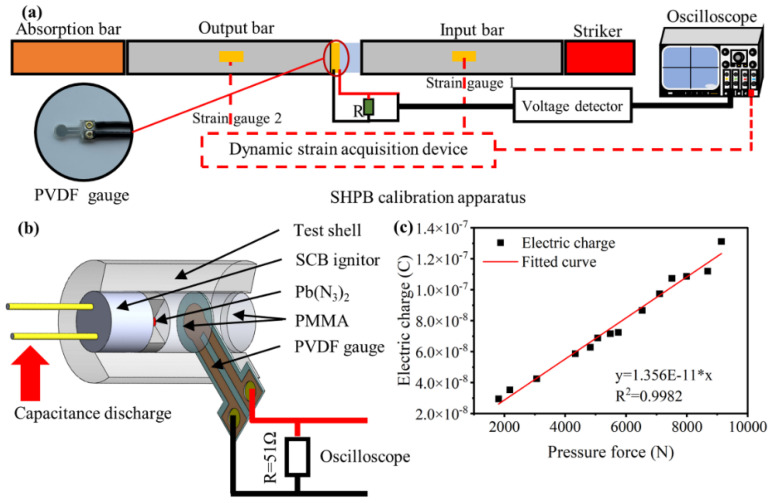
(**a**) Schematic diagram of SHPB calibration apparatus. (**b**) PVDF gauges test circuit. (**c**) Electric charge obtained by PVDF gauges as a function of pressure force generated by SHPB.

The PVDF gauges with the effective area of 0.1018 cm^2^ were sandwiched between input and output bars as shown in Figure 2a. Upon impact, the pressure force transmitting through the PVDF was calculated with strain gauges mounted on the bars. Meanwhile, the electric charge generated from PVDF gauges was recorded by a digital oscilloscope with a test circuit as shown in Figure 2b. Due to the linear relationship between the pressure force and charge amount in PVDF within a large range, the dynamic piezoelectric coefficient of PVDF gauges was determined according to Equation (3), which is expressed as

(3) e=kF

where e represents the electric charge amount generated by PVDF gauge under the pressure force, F represents the pressure force, and k represents the dynamic piezoelectric coefficient of PVDF gauges.

The dynamic piezoelectric coefficient of PVDF gauges is equal to 13.56 pC/N by fitting, as shown in Figure 2c.

After the PVDF gauges are calibrated, the shockwave attenuation coefficient of PMMA can also be determined. The output pressure of the LA microcharge can be calculated by Equations (4) and (5), which are expressed as

(4) $$P_{out}=ake_{out}$$

(5) $$a=\frac{P_{s}}{ke_{s}}$$

where $P_{out}$ represents the output pressure of LA microcharges, k represents the calibrated dynamic piezoelectric coefficient of PVDF gauges, $e_{out}$ represents the charge amount of LA measured in pressure test, a represents the correction factor of the test, $P_{s}$ represents the steady detonation pressure of the LA microcharge (17.12 GPa, calculated by steady detonation velocity and particle velocity of LA micro charge), and $e_{s}$ represents the charge amount in the PVDF gauge test generated by steady detonation of LA microcharges.

Through the combination of confinements with different heights, the output pressure of LA microcharges at the heights of 0.5, 0.8, 1.0, 1.3, 1.5, 1.8, 2.0, 2.3, 2.5, 3.0, and 4.0 mm were measured. Pressure data were obtained at each height with a minimum of three shots.

### 2.3. Particle-Velocity Test

**Figure 3:**
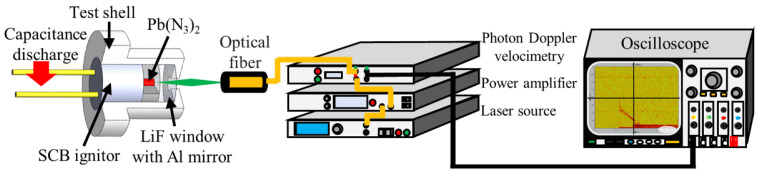
Schematic of test sample and PDV system.

The use of PDV is a well-established approach in study of shock compression and detonation wave profiles [20,21,22,23,24,25]. Herein, for experimental consistency and subsequent calculations, the particle-velocity profiles of the LA/LiF window interface at the heights chosen in the PVDF pressure tests were performed by using PDV, as depicted in Figure 3. 

**Figure 4:**
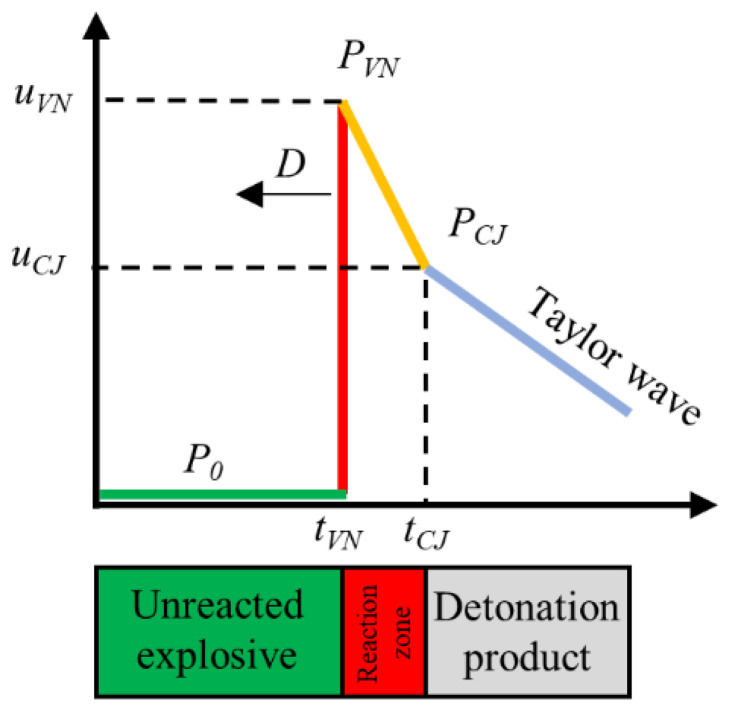
Typical particle-velocity profile (**above**) and schematic of ZND model (**below**).

The ZND model, as shown in Figure 4, for steady detonation of near-ideal high explosives, predicts two crucial states in detonation structure: the von Neumann spike (*VN* spike), which corresponds to the front shock wave, and the *CJ* state (*CJ* point) that defines the end of the reaction or the beginning of Taylor expansion zone, wherein the energy release from the detonation reactions is completed and coincides with the sonic plan [26,27]. Therefore, the stable velocity of the end of the detonation–reaction interface is considered to be the steady detonation velocity. In data processing, the particle velocity of the *VN* spike was read directly from the velocity curve, and the particle velocity of the *CJ* point is regarded as the inflection point on the particle-velocity curve, according to many studies.

In the PDV test, due to the effect of the shockwave, the refractive index of the LiF window will change. Therefore, all of the particle velocities given in this research have been corrected with Equation (6), expressed as

(6) $$u_{s}=\frac{u}{1.2678}$$

where $u_{s}$ represents the true particle velocity, u represents the measured velocity, and 1.2678 is the window correction of the refractive index of a shocked LiF single crystal at 1550 nm wavelength.

Because of the similar impedance between detonation products and the LiF window, an acoustic approximation method was commonly used to calculate the pressure in the detonation zone [28]. This method is expressed as

(7) $$P=\frac{1}{2}u_{s}\left(\rho_{w0}\left(C_{w0}+\lambda u_{s}\right)+\rho_{e0}D\right)$$

where P represents the detonation pressure in the detonation-reaction zone, $\rho_{w0}$ represents the density of the LiF window, $C_{w0}$ and λ represent the shock Hugoniot parameters of the LiF window, $\rho_{e0}$ represents the density of explosive, and D represents the detonation velocity of the explosive.

Additionally, the detonation-reaction width and time were determined by Equations (8) and (9), expressed as

(8) $$b=\int_{0}^{\tau }\left(D-u_{s}\right)dt$$

(9) $$\tau =t_{CJ}-t_{VN}$$

where b represents the detonation-reaction zone width, τ represents the detonation-reaction time, and $t_{CJ}$ and $t_{VN}$ represent the time of the *CJ* point and *VN* spike in particle-velocity profiles.

With steady detonation velocity and the particle velocity of *CJ* point, the *CJ* pressure of LA charges with a diameter of 1.2 mm was calculated for the correction factor in Equation (5).

## 3. Results and Discussion

### 3.1. Steady Detonation Velocity of LA Microcharge

**Figure 5:**
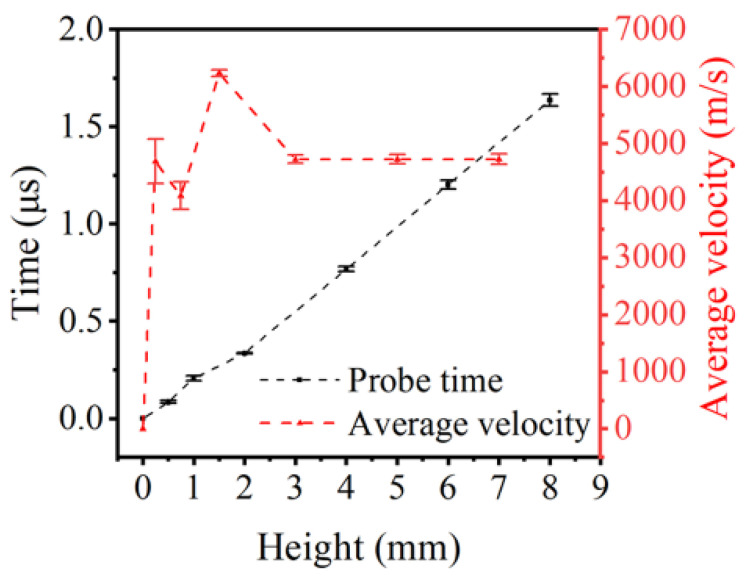
Probes signal time as a function of charge height and average velocity as a function of charge height.

The average detonation velocities at the heights of 0.25, 0.75, 1.5, 3.0, 5.0, and 7.0 mm were calculated by Equations (1) and (2) and shown in Figure 5. The graph reveals that the average velocity has a sharp rise at the height of 0~1.5 mm, then reaches a peak of 6233 m/s at approximately 2.0 mm, and finally decays at a plateau of 4726 ± 8 m/s by 3.0 mm. In contrast to the results obtained in large pellets, a significant drop of velocity was observed in small charges. Another interesting aspect of this graph is the steady detonation velocity of LA with a diameter of 1.2 mm, measured—not calculated—which provides the basis for subsequent calculations. Hence, the detonation process of LA can be roughly described as follows. First, within the height of 3.0 mm, the detonation propagates unsteadily after ignition. Second, after 3.0 mm, the steady detonation has formed. However, the figure cannot provide the precise critical height between unsteady and steady detonation, due to the limitation of the probe size.

### 3.2. Output Pressure of LA Microcharge

**Figure 6:**
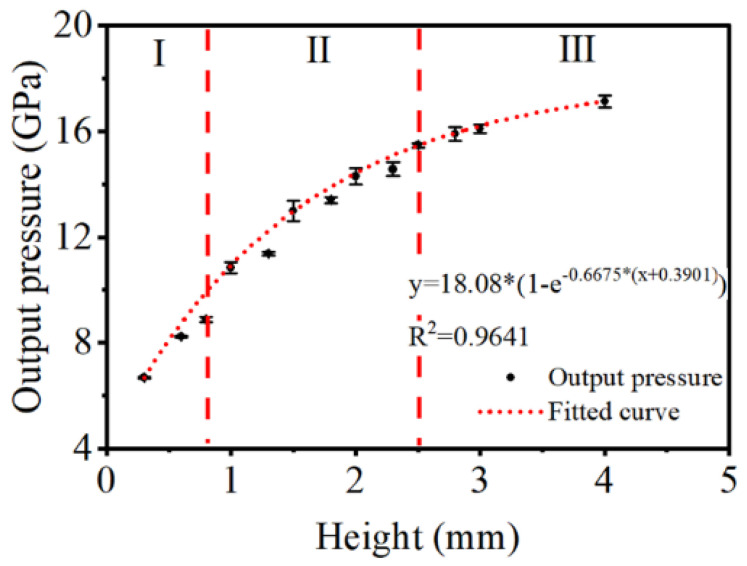
Output pressure as a function of charge height.

The figure below illustrates the main results of the intercorrelation between output pressure and charge height. The output pressure increases logarithmically with the height and is projected to remain steady at 17~18 GPa. Closer inspection of Figure 5 and Figure 6 show that both velocity and pressure share the same height of reaching stability at approximately 2.5~3.0 mm. 

In the pressure experiment, after reaching the interface between the LA and PMMA barrier, the front shockwave will decay rapidly and be caught up by the detonation reaction as a consequence of no reaction energy release, which sustains a steady shock profile in PMMA. Therefore, the pressure measured by PVDF gauges corresponds to the *CJ* pressure, or the maximum pressure generated by detonation reactions. 

Taken together, according to data in Figure 5 and Figure 6, we can provide further descriptions of the detonation processes of LA microcharges. First, the detonation reaction starts to accelerate at 0~0.8 mm, as both velocity and pressure rise with height. Second, the detonation attempts to reach a steady state, for there is a significant difference of trend between velocity and pressure. Namely, the velocity peaks at ~1.5 mm, while the pressure increases steadily. Third, stable detonation is formed, as velocity and pressure level off after ~2.5 mm.

### 3.3. Particle Velocity Profile of LA Microcharge

**Figure 7:**
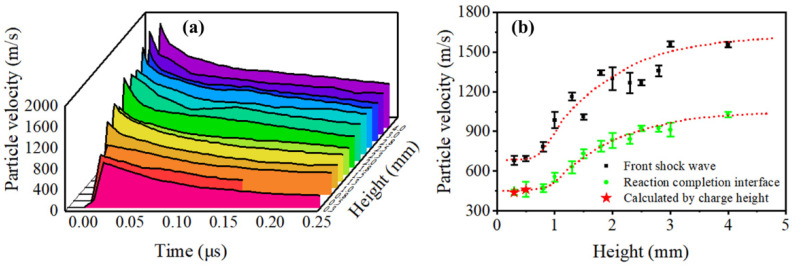
(**a**) Particle-velocity profiles at the heights of 0.3~4.0 mm. (**b**) Particle velocity of the shock front and reaction–completion interface as function of charge height.

Figure 7a presents an overview of all the particle-velocity profiles at the heights of 0.3, 0.5, 0.8, 1.0, 1.3, 1.5, 1.8, 2.0, 2.3, 2.5, 2.8, 3.0, and 4.0 mm, and each curve is the average of three or more shots. The shape of the profiles is similar to the profile predicted by the ZND model. Slight differences, however, were found between profiles with larger and smaller heights. According to the discussion above, for steady detonations, such as the LA charges with the heights of 2.5, 2.8, 3.0, and 4.0 mm, the shock front (*VN* spike) and *CJ* point can be clearly defined and read in the particle-velocity profiles. As for unsteady detonations, such as profiles with lower heights, the shock fronts are not obvious. Interestingly, we can still find the exact point or interface between the reaction zone and Taylor zone in profiles, which does not represent the *CJ* state but merely the end of detonation reactions. Moreover, the velocity of this interface indicates the propagation of an unsteady detonation reaction directly. Thus, in this paper, both interfaces between the reaction zone and Taylor zone in steady or unsteady detonations will refer to the reaction–completion interface. 

The particle velocity of the *VN* spike and reaction–completion interface in tests is collected and shown in Figure 7b. The error bars are also given. For unsteady detonation within the height of 0.8 mm, the particle velocity of the reaction–completion interface was obtained by iterative calculations with charge height. Both particle velocity of the reaction–completion interface and the front shockwave increase significantly at the height between 0.8 mm and 2.5 mm, and are expected to remain steady after 3.0 mm, which is in line with results discussed in Figure 5 and Figure 6. The data fluctuation of shock front velocity is more obvious than the experimental data of the reaction–completion interface, stemming from the possible density distribution in microcharges, which is hard to control. It is noteworthy that the particle velocity of the shock front remains unchanged within the height of 0.8 mm, indicating that the initial shock wave does not decay. Furthermore, the particle velocities of the reaction–completion interface within the height of 0.8 mm are also approximately the same.

**Figure 8:**
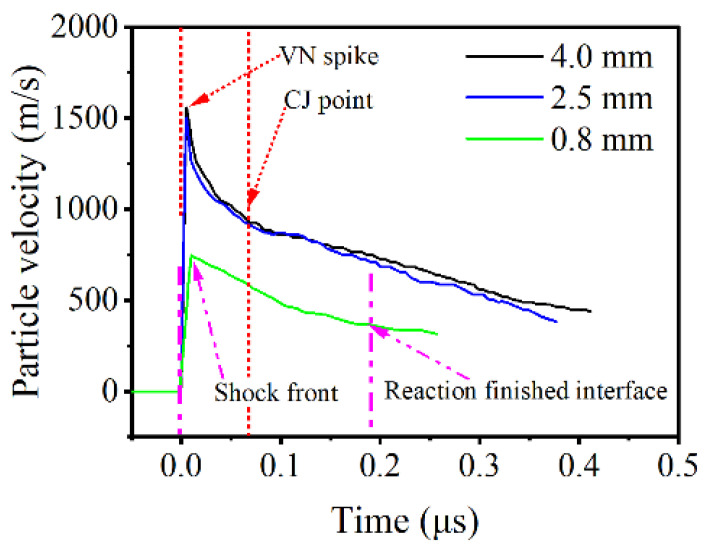
Detailed particle-velocity profiles at the heights of 4.0, 2.5, and 0.8 mm.

Figure 8 compares the particle-velocity profiles of 0.8 mm, 2.5 mm, and 4.0 mm. The particle-velocity profile at 0.8 mm does not have a *VN* spike and obvious reaction–completion interface, because the energy released in reactions is not enough to support a steady shock profile. The particle-velocity profiles at 2.5 mm and 4.0 mm have the same particle velocity of the *VN* spike and *CJ* point, which indicates that the detonation is stable at 2.5 mm.

Taken together, the reaction of LA within the height of 0.8 mm can only be considered as volume explosion, as discussed in previous work [1], for lack of an obvious interface between reaction zone and product-expansion zone and complete detonation profile.

### 3.4. Detonation Propagation of LA Micro Charge

The velocity of the detonation reaction–completion interface represents the detonation propagation directly, but it is difficult to measure. To solve this problem, we combined the PVDF and PDV data to calculate the velocity of the reaction–completion interface. Equation (7) mentioned above is actually a one-dimensional approximate result to the shockwave [26,27]. The detonation pressure or *CJ* pressure can be determined with a known particle velocity of *CJ* point and steady detonation velocity. Therefore, for unsteady detonation, the velocity of the reaction–completion interface can be calculated by Equation (8), when the output pressure and the particle velocity of the reaction–completion interface are known. This equation is expressed as

(10) $$D^{\prime }=\frac{\frac{2P^{\prime }}{{u_{s}}^{\prime }}-\rho_{w0}\left(C_{w0}+\lambda {u_{s}}^{\prime }\right)}{\rho_{e0}}$$

where $D^{\prime }$ represents the velocity of the reaction–completion interface in unsteady detonation, $P^{\prime }$ represents the output pressure, and ${u_{s}}^{\prime }$ represents the particle velocity of the reaction–completion interface. 

**Figure 9:**
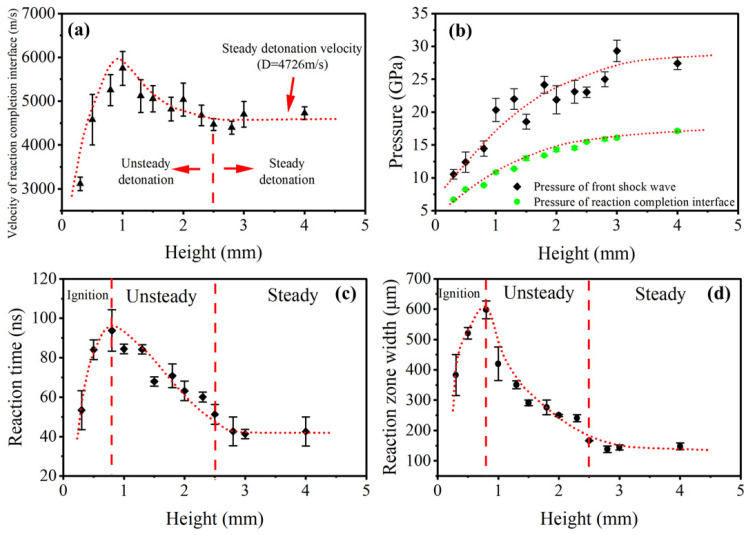
(**a**) Velocity of detonation-reaction–completion interface calculated based on experimental data. (**b**) Pressure of shock front (calculated) and pressure of the reaction–completion interface (experimental data from PVDF gauges) as a function of charge height. (**c**) Detonation-reaction zone duration as a function of charge height. (**d**) Detonation-reaction zone width as a function of charge height.

The pressure and particle velocity of the interface are much easier to measure compared with the probe test at microscale. If the PDV and pressure measurement apparatus are combined, the basic parameters in micro detonation of various energetic materials can be obtained in one shot. The main results of the detonation growth of the LA microcharge are summarized in Figure 9.

As shown in Figure 9a, the trend of the calculated interface velocity is similar to the results in the probe tests. The interface velocity has a sharp rise at 0~1.0 mm, then reaches a peak of 5750 m/s at 1.0 mm, and finally levels off after 2.5 mm. As a point of reference, the steady detonation velocity in the literature (4860 m/s, 3.571 g/cm^3^ [29]) is larger than the steady detonation velocity in tests (4726 m/s, 3.571 g/cm^3^), which is attributed to the more energy dissipation at small geometries, also known as the diameter effect. 

The pressure of the shock front was calculated based on the particle velocity of the front shock wave and the velocity of the reaction–completion interface which was corrected by local particle velocity, as shown in Figure 9b. The pressure of the shock front is much higher than the pressure of the reaction interface, as a result of the enhancement of chemical energy released in the reaction zone. In addition, the data of shock front pressure fluctuates more than the data of reaction interface pressure, which proves that the shock front is more susceptible to initial density of charges. Both pressure of the shock front and the reaction interface increase with height, and are expected to remain steady after 2.5 mm at 27.42 ± 1.02 GPa and 17.12 ± 0.22 GPa.

Figure 9c,d shows the detonation–reaction zone duration and width calculated based on experimental data. For the data at 0.3 mm and 0.5 mm, the reaction-zone width was determined by the total height of microcharges, as was the reaction time. The reaction-zone time and width in LA charges with a diameter of 1.2 mm peaks at 0.8~1.0 mm, then decays at a plateau of 44 ± 7 ns and 148 ± 11 μm by 2.5 mm. It is worth noting that the reaction zone width within 0.8 mm is smaller than the corresponding height, which proves that the LA cannot form a steady detonation profile within 0.8 mm as discussed above.

In most high explosives, the detonation velocity increases with height and density within a certain range. However, several differences have been found in the detonation of LA charges with regard to small height. A peak of detonation velocity appears at 0.8~1.0 mm after the ignition, which is also higher than the steady detonation velocity at macroscale. The density of an unreacted area in LA increases significantly because of the precompression of the front shock wave, which leads to a high reaction ratio in the compressed area. Both the high density and reaction ratio lead to a sharp rise in velocity. Moreover, the reaction within 0.8 mm is the volume reaction for front shock or reaction-particle velocity profiles, which have not changed significantly. Therefore, the whole charge can be considered as the reaction zone. 

After the detonation reaction reaches 0.8 mm, the completed detonation structure is well-formed. Then the shock front, reaction zone, and detonation products’ expansion zone will try to stabilize at the external conditions. Based on the stability of shock profiles, the reaction zone cannot overtake the shock front even if the reaction rate can achieve an extreme value decided by local density and pressure. The detonation structure actually limits the velocity of the reaction–completion interface, because the velocity of the shockwave is determined by the state parameters of local material associated with high pressure in the reaction zone. On the one hand, the high pressure intensifies the reactions in detonation. On the other hand it also increases the velocity of the shockwave, showing the dynamic processes of the shock front and reaction zone. It is quite interesting that the reaction wave is catching up to the front shockwave, although the velocity of the reaction–completion interface tapers off to a stable value. The whole detonation profile is under the balance of thermodynamics and kinetics with external conditions when the detonation reaches the certain height of 2.5 mm.

## 4. Conclusions

Based on the detonation parameters obtained from experiments or calculated, a detailed description of three phases in the detonation propagation of LA microcharges with a diameter of 1.2 mm can be given. First, the volume reaction is carryied out within 0.8 mm after the initial shockwave. Second, the complete detonation profile sustained by the chemical energy of the reaction zone is formed at 0.8~2.5 mm. In addition, the reaction zone catches up to the shock front, leading to the compression of the whole structure. Third, a steady detonation profile occurs at 2.5 mm stemming from the balance of the reaction zone and shock front. The detonation growth phenomenon in microcharges is not clear enough. More work shall be done to fully understand the detonation mechanism at microscale. The method we used in this paper will generate fresh insight for researches in various nonideal energetic materials with small geometries.

## Data Availability

Data available on request, having regard to restrictions, e.g., privacy or ethical.
